# Flax (*Linum usitatissimum* L.) response to non-optimal soil acidity and zinc deficiency

**DOI:** 10.1186/s12870-019-1641-1

**Published:** 2019-02-15

**Authors:** Alexey A. Dmitriev, George S. Krasnov, Tatiana A. Rozhmina, Alexander V. Zyablitsin, Anastasiya V. Snezhkina, Maria S. Fedorova, Elena N. Pushkova, Parfait Kezimana, Roman O. Novakovskiy, Liubov V. Povkhova, Marina I. Smirnova, Olga V. Muravenko, Nadezhda L. Bolsheva, Anna V. Kudryavtseva, Nataliya V. Melnikova

**Affiliations:** 1 Engelhardt Institute of Molecular Biology, Russian Academy of Sciences, Moscow, Russia; 2 All-Russian Research Institute for Flax, Torzhok, Russia

**Keywords:** Flax, *Linum usitatissimum*, Non-optimal acidity, Zinc deficiency, Stress response, High-throughput sequencing

## Abstract

**Background:**

Flax (*Linum usitatissimum* L.) is grown for fiber and seed production. Unfavorable environments, such as nutrient deficiency and non-optimal soil acidity, decrease the quantity and quality of yield. Cultivation of tolerant to stress varieties can significantly reduce the crop losses. Understanding the mechanisms of flax response to the stresses and identification of resistance gene candidates will help in breeding of improved cultivars. In the present work, the response of flax plants to increased pH level and zinc (Zn) deficiency was studied.

**Results:**

We performed high-throughput transcriptome sequencing of two flax cultivars with diverse tolerance to increased pH level and Zn deficiency: Norlin (tolerant) and Mogilevsky (sensitive). Sixteen cDNA libraries were created from flax plants grown under control conditions, increased pH level, Zn deficiency, and both stresses simultaneously, and about 35 million reads were obtained for each experiment type. Unfavorable pH resulted in significantly stronger gene expression alterations compared to Zn deficiency. Ion homeostasis, oxidoreductase activity, cell wall, and response to stress Gene Ontology terms were the most affected by unfavorable pH and Zn deficiency both in tolerant and sensitive flax cultivars. Upregulation of genes encoding metal transporters was identified under increased pH level, Zn deficiency, and both stresses simultaneously. Under Zn deficiency, only in tolerant cultivar Norlin, we revealed the induction of several photosynthesis-related genes and, in this way, this tolerant genotype could overcome unfavorable effects of reduced Zn content.

**Conclusions:**

We identified genes with expression alterations in flax under non-optimal soil acidity and Zn deficiency based on high-throughput sequencing data. These genes are involved in diverse processes, including ion transport, cell wall biogenesis, and photosynthesis, and could play an important role in flax response to the studied stresses. Moreover, genes with distinct expression changes between examined tolerant and sensitive genotypes could determine the mechanisms of flax tolerance to non-optimal soil acidity and Zn deficiency.

**Electronic supplementary material:**

The online version of this article (10.1186/s12870-019-1641-1) contains supplementary material, which is available to authorized users.

## Background

Flax (*Linum usitatissimum* L.) is grown for fiber and seed production and used in textile, pharmaceutical, food, paint, and varnish industries [1]. Unfavorable environments, such as nutrient deficiency and non-optimal soil acidity, decrease the quantity and quality of flax yield. Optimal pH level for flax growing is about 5.0–5.5 [2], however, excessive application of lime results in an increase of pH and imbalance of macro- and microelements in soil (especially zinc (Zn) deficiency) that causes physiological depression of flax plants [3]. *L. usitatissimum* genotypes differ in their tolerance to increased soil pH (7.5 or higher) and Zn deficiency and cultivation of varieties that are tolerant to the stresses can significantly reduce the crop losses [2]. Understanding the mechanisms of flax response to increased pH and Zn deficiency and identification of resistance gene candidates will help in breeding of improved cultivars.

High-throughput sequencing is intensively used for studying flax response to diverse stresses, including drought [4], alkalinity and salt [5], excess concentration of aluminum ions [6–8], imbalanced nutrition [9–11], *Fusarium oxysporum* infection [12, 13]. These studies allowed the revelation of genes with expression alterations under the stresses and identification of processes on which the unfavorable conditions have the greatest impact. Alterations in gene expression under strong alkaline stress (pH 11.6) were investigated in flax plants and genes related to response to biotic stimulus were found to be particularly affected by alkalinity, while photosynthesis-related genes were affected by combined alkaline-salt stress [14]. However, transcriptome studies of flax plants under the close to natural conditions of increased pH level (7.5) were not performed and the influence of Zn deficiency on gene expression in flax was not investigated, although, for other plants species, such works aimed at identification of differentially expressed genes were carried out [15–20]. Zn deficiency leads to disruption of enzyme activity, inhibition of photosynthesis, production of reactive oxygen species (ROS), and increased iron accumulation [21, 22]. Zn chelators, including nicotianamine, phytosiderophore, glutathione, phytochelatin [23–26], and transporters, including ZRT (Zinc-Regulated Transporter) - IRT (Iron-Regulated Transporter) - like proteins (ZIPs), Natural Resistance-Associated Macrophage Proteins (NRAMPs), Yellow Stripe-Like (YSL) proteins, Cation Diffusion Facilitator (CDF), heavy metal tolerance (HMA) proteins [27–34], are involved in Zn homeostasis in plants. Alkaline stress disrupts uptake of metal micronutrients, including Zn and Fe, induced production of ROS, and results in alterations in levels of antioxidants, transcriptional factors, phytosiderophores, nicotianamine, photosynthesis-related and heat shock proteins in plants [35–44].

At the present study, we performed high-throughput transcriptome sequencing of flax plants grown under increased pH level, Zn deficiency, and both stresses simultaneously and evaluated gene expression alterations to determine the negative effects of these factors. Since the comparison of responses to the stress for tolerant and sensitive genotypes of the same species is especially important for identification the tolerance mechanisms, we used two flax genotypes with diverse tolerance.

## Methods

### Plant material

*L. usitatissimum* cultivars, Norlin and Mogilevsky, which are respectively tolerant and sensitive to unfavorable for flax increased pH level of soil [7], were chosen for the present study. Flax seeds were sterilized with 1% sodium hypochlorite solution for 10 min and then germinated on filter paper in Petri dishes under sterile conditions. Two days later, the germinated seeds were transferred to 15 ml tubes with Murashige-Skoog medium or its modifications with different pH and zinc content: 1) control: pH = 5.5, standard zinc content – 8.6 mg/l ZnSO_4_·7H_2_O (pH = 5.5, Zn+); 2) pH = 7.5, standard zinc content (pH = 7.5, Zn+); 3) pH = 5.5, 1000-fold reduced Zn content (pH = 5.5, Zn-); 4) pH = 7.5, 1000-fold reduced Zn content (pH = 7.5, Zn-). About 200 plants of Norlin (tolerant) and Mogilevsky (sensitive) cultivars were grown in a climate chamber for 3 weeks, after which the root tips about 2 cm in length were collected and frozen in liquid nitrogen followed by storage at − 70 °C.

### High-throughput sequencing

Total RNA was isolated from roots using RNeasy Plant Mini Kit (Qiagen, USA). In total, 32 RNA samples were isolated from pools of 4–6 plants: two cultivars in four experiment conditions, four biological replicates. The concentration and quality of the isolated RNA were assessed using Qubit 2.0 fluorometer (Life Technologies, USA) and Agilent 2100 Bioanalyzer (Agilent Technologies, USA). For the preparation of cDNA libraries for transcriptome sequencing, two RNA samples from plants of the same cultivar grown under identical conditions were combined in equimolar amounts. As a result, 16 pools of RNA samples were obtained. To prepare the cDNA libraries for high-throughput sequencing, the TruSeq Stranded Total RNA Sample Prep Kit (Illumina, USA) was used. Sixteen cDNA libraries were obtained, the quality of which was evaluated using Agilent 2100 Bioanalyzer (Agilent Technologies). The average length of the cDNA libraries was 270 nucleotides; the adapter dimers were absent; library concentrations were about 13–25 ng/μl. Obtained libraries were mixed in equimolar concentrations and sequenced on NextSeq500 sequencer (Illumina) with 80-nucleotide read length.

### Data analysis

First, we removed adapters and trimmed reads with trimmomatic (TRAILING:30 SLIDINGWINDOW:4:20 ILLUMINACLIP:<file>:2:30:10:8:TRUE) [45]. Next, we removed vectors and contaminants using Kraken [46] based on the MiniKraken DB. The assembly of transcripts was performed using Trinity 2.4.0 software with the default parameters [47]. We carried out both genome-guided and non-guided assemblies separately for each flax cultivar and for both studied cultivars together. For genome-guided assembly, we used *Linum usitatissimum* reference genome GCA_000224295.2/ASM22429v2 (the total length is 316 Mb). Next, we filtered out transcripts shorter than 500 bp. The quality of transcriptome assemblies was evaluated using ExN50 statistics (Trinity) and the analysis of the presence of highly conservative single-copy orthologues – with BUSCO [48]. The transcripts and their predicted proteins were annotated using Trinotate pipeline (http://trinotate.github.io/). The resulting transcript sequences were analyzed for the presence of ORF using TransDecoder [49]. Transcripts and proteins were analyzed using the UniProt database by blastx and blastp respectively. Protein sequences were analyzed for the presence of PFAM domains with HMMER [50, 51]. Transcripts and proteins were annotated with Gene Ontology (GO), KEGG, and COG databases.

Obtained reads were mapped to the assembled transcripts using bowtie2 [52] and RSEM [53] and read counts per each transcript were determined. The read counts were analyzed using the edgeR software [54]. Transcripts with a counts per million (*CPM*) less than 2 were filtered out. Multidimensional scaling plot of distances between gene expression profiles in plants of two flax cultivars under the studied conditions was created with edgeR [54]. To evaluate expression alterations of each transcript for tolerant and sensitive cultivars, log_2_(fold change) values were calculated. False discovery rate (FDR) values were obtained by Benjamini-Hochberg *p*-value adjustment. The gene set enrichment analysis (GSEA) was performed using Goseq (http://bioconductor.org/packages/release/bioc/html/goseq.html), in which top lists of upregulated or downregulated genes were used. Visualization of gene expression alterations in selected GO terms was performed by heatmaps created using edgeR [54].

## Results

### Transcriptome sequencing and annotation

To study the response of flax plants to non-optimal soil acidity and zinc deficiency, cultivars with different tolerance to the stress were selected on the basis of our previous investigations: tolerant Norlin and sensitive Mogilevsky [7]. About 200 plants were grown under control conditions, Zn deficiency (1000-fold reduced Zn content), increased pH level (7.5), and both Zn deficiency and pH = 7.5. After 3 weeks, the phenotype assessment of plants was carried out before collecting material for the sequencing. We observed moderate inhibition of plant growth, leaf bronzing, yellow and necrotic spots on leaves under Zn deficiency in both cultivars. Under increased pH level, growth inhibition and necrosis were more pronounced, especially in sensitive cultivar Mogilevsky. Under both increased pH level and Zn deficiency, strong inhibition of plant growth and necrosis were observed in studied cultivars, however, the symptoms were especially pronounced in Mogilevsky.

We collected plant roots for RNA extraction and prepared cDNA libraries for transcriptome sequencing in duplicate for each cultivar under each of the studied conditions. In total, 16 cDNA libraries were sequenced on the Illumina platform and from 28 to 41 million 80-nucleotide reads were obtained for each sample.

**Fig. 1:**
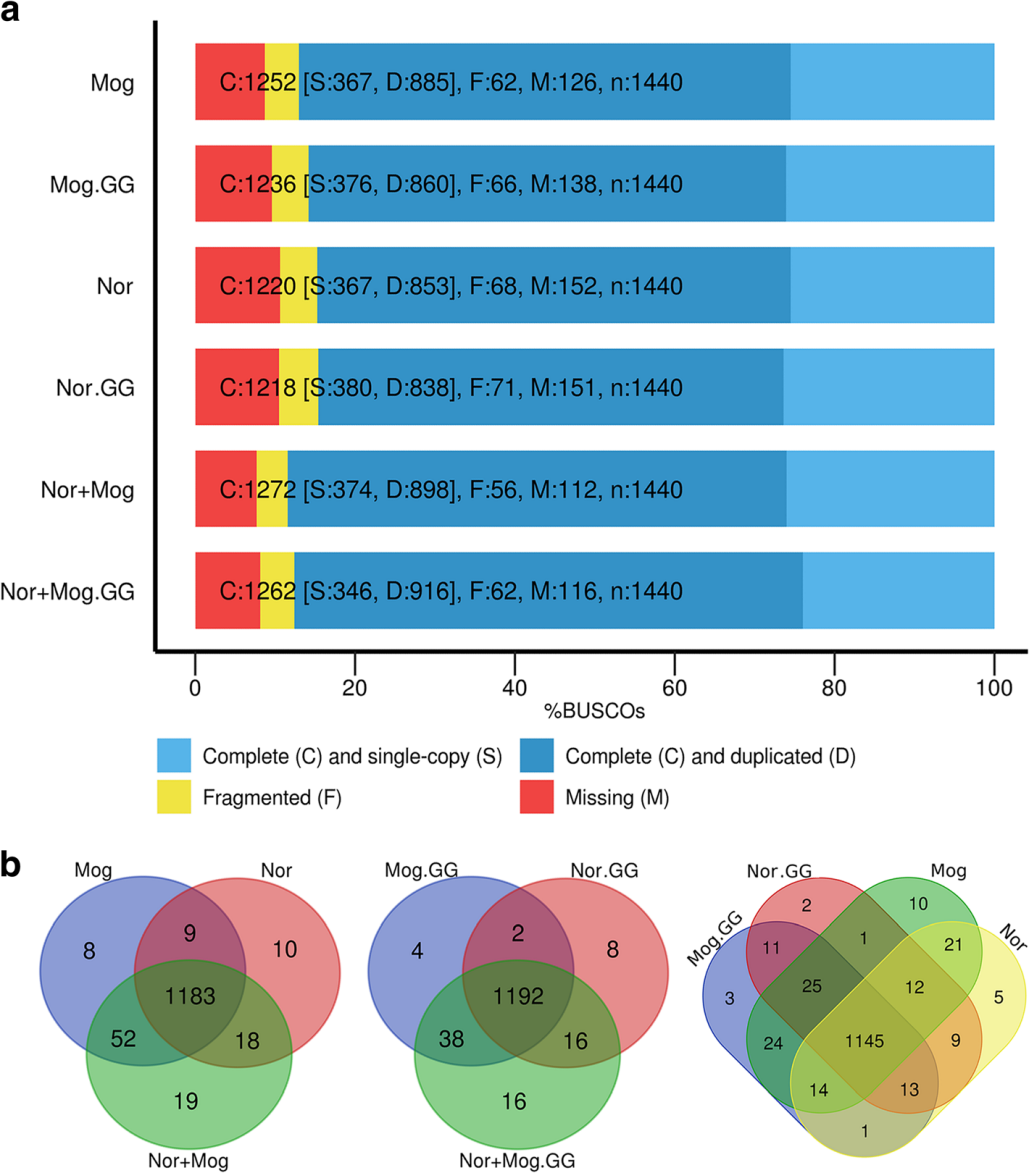
Assessment of the completeness of the transcriptome assemblies using BUSCO. Transcripts longer than 500 bp were taken in account. The upper part of figure (**a**) demonstrates the results of completeness evaluating of the transcriptome assemblies using BUSCO. The bottom part of figure (**b**) presents three Venn diagrams that illustrate overlaps of the lists of complete BUSCOs between the assemblies. GG – genome-guided assemblies. Mog – cultivar Mogilevsky, Nor – cultivar Norlin, Nor+Mog – mixed dataset of Norlin and Mogilevsky cultivars

Then, transcriptome assemblies were performed for each cultivar and for the mixed dataset, both genome-guided and non-guided. Genome-guided (GG) assemblies had a bit smaller total length, a greater number of genes but less number of transcripts (Table 1). N50 was slightly greater for GG assemblies. Next, we evaluated the completeness of the transcriptome assemblies with BUSCO. Here, GG assemblies did not demonstrate any advantages over non-guided ones. Moreover, non-guided assemblies had a slightly greater number of complete BUSCOs (Fig. 1).

**Table 1:** Transcriptome assembly statistics for flax cultivars (transcripts > 500 bp)

Assembly	Non-guided			Genome-guided		
Cultivar	Norlin	Mogilevsky	Norlin + Mogilevsky	Norlin	Mogilevsky	Norlin + Mogilevsky
Total length, Mb	105.7	106.8	124.4	100.7	103.1	122.9
Transcripts	72,885	72,488	84,428	67,565	68,250	78,663
Genes	26,228	25,833	30,532	41,326	41,191	46,444
Average length	1450	1474	1473	1491	1511	1562
N50	1707	1738	1763	1774	1796	1883
GC %	45.1	45.2	45.0	44.6	44.7	44.4
Complete^a^ BUSCOs	1220	1252	1272	1218	1236	1262
Missing BUSCOs	152	126	112	151	138	116
Fragmented BUSCOs	68	62	56	71	66	62

^a^ – Single-copy or duplicated complete BUSCOs

The transcriptome assemblies derived for the mixed dataset (Norlin and Mogilevsky cultivars) demonstrated a slight advantage over the individual assemblies in terms of the number of complete BUSCOs (2–4% greater) both for GG and non-guided assemblies. Wherein, the number of genes/transcripts and the total transcriptome length differed dramatically (15–25%). This indicates the presence of orthologous alleles (coming from each cultivar) in the mixed assembly for about 15–25% genes/transcripts. Therefore, we did not use mixed assemblies in the further gene expression analysis between two cultivars because reads from orthologous alleles will be mapped to their own targets in the assembly that will lead to the false-positive differential expression. Hence, for the further analysis, we used non-guided assembly of tolerant cultivar Norlin. Additionally, we checked the completeness of transcriptome assembly containing transcripts > 50 bp (Trinity’s default value) and showed that the number of complete BUSCOs did not increase compared to the assembly containing transcripts > 500 bp, which was used in the present study.

About 90.0–90.4% of Mogilevsky reads and 90.3–90.9% of Norlin reads were successfully mapped to the Norlin assembly using bowtie2 (Additional file 1). As can be seen, despite the genetic divergence between the cultivars, a single transcriptome assembly may be correctly used for gene expression quantification.

### Expression alterations

**Fig. 2:**
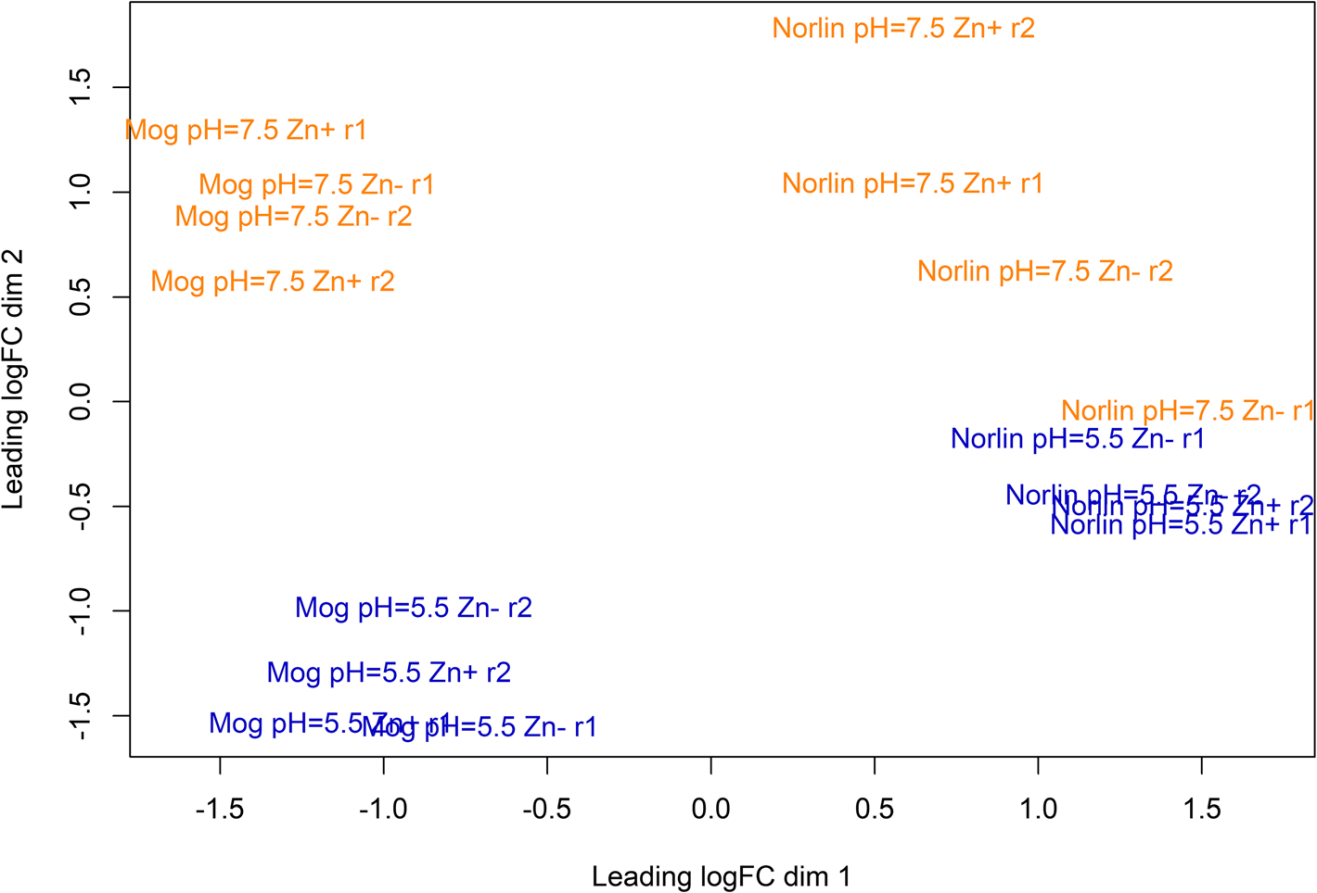
Multidimensional scaling plot of distances between gene expression profiles of two flax cultivars under the studied conditions. The distance between samples indicates their diversity. Cultivars are designated Mog (Mogilevsky) and Norlin, r1 and r2 – biological replicates. Control conditions are indicated pH = 5.5 Zn+; optimal pH level and Zn deficiency – pH = 5.5 Zn-; increased pH level and optimal Zn content – pH = 7.5 Zn+; increased pH level and Zn deficiency – pH = 7.5 Zn-. Blue color – pH = 5.5, orange color – pH = 7.5

For visualization of the differences in expression profiles between samples, multidimensional scaling (MDS) plot was created (Fig. 2). As seen from Fig. 2, the samples of Mogilevsky cultivar were separated into two groups: Mogilevsky under pH = 5.5 and Mogilevsky under pH = 7.5. For Norlin cultivar, the separation on the basis of pH level was also observed, but it was not so clear as for Mogilevsky cultivar: one of the biological replicates of “pH = 7.5 Zn-” conditions was close to the subgroup of Norlin under pH = 5.5. We suggest that this discordance of replicates was due to biological variability of plants taken in cDNA library preparation. Nevertheless, it can be concluded that samples of each cultivar under the same pH had close gene expression profiles regardless of Zn content. Thus, unfavorable pH level had a greater effect on flax gene expression compared to Zn deficiency. Cultivar-specific differences in expression profiles were also observed under all studied conditions and samples of the same cultivar were grouped together.

**Fig. 3:**
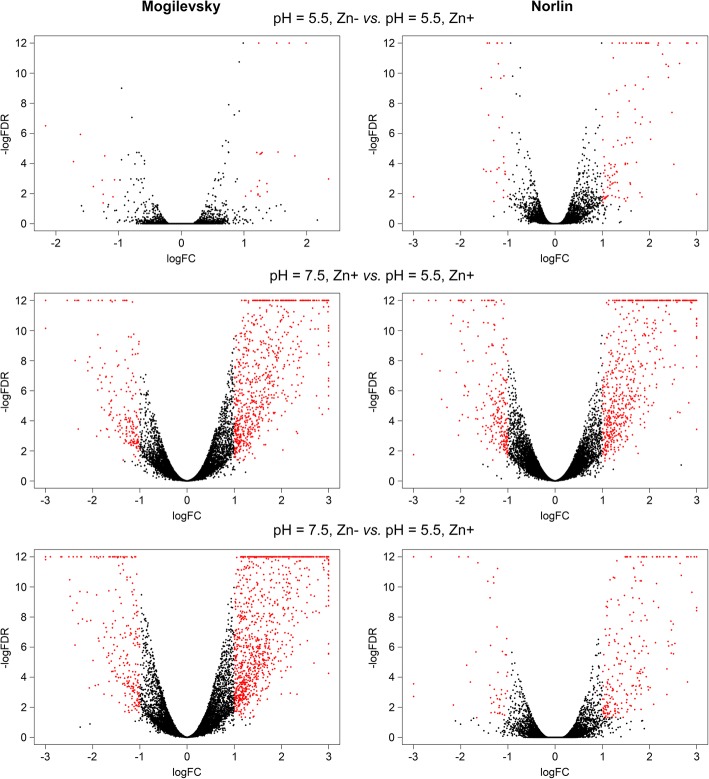
Expression alterations in two flax cultivars under Zn deficiency, unfavorable pH, and both stresses. Volcano plots illustrate the results of differential expression analysis induced by Zn deficiency (pH = 5.5, Zn-), unfavorable pH (pH = 7.5, Zn+), and both stresses simultaneously (pH = 7.5, Zn-) in flax cultivars Norlin (tolerant) and Mogilevsky (sensitive). Each point represents one gene. LogFC – binary logarithm of expression level fold change; LogFDR – decimal logarithm of the false discovery rate. Genes with expression fold change (increase or decrease) > 2 and FDR < 0.05 are marked with red

**Fig. 4:**
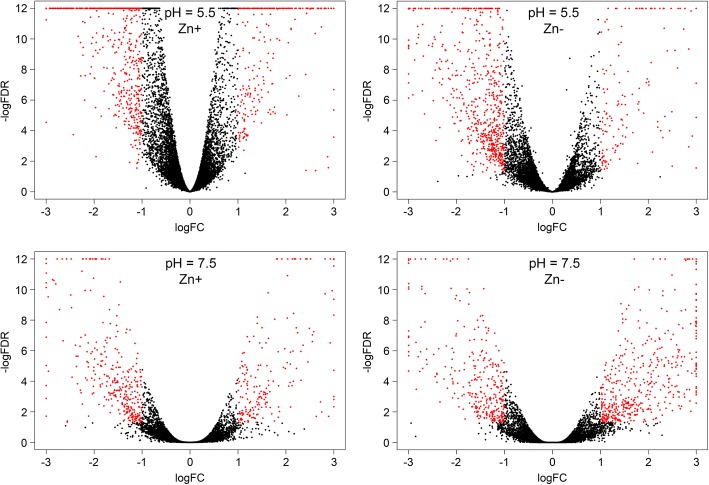
Expression differences in flax cultivars Norlin and Mogilevsky. Volcano plots illustrate the results of differential expression analysis between flax cultivars Norlin (tolerant) and Mogilevsky (sensitive) under control conditions (pH = 5.5, Zn+), Zn deficiency (pH = 5.5, Zn-), unfavorable pH (pH = 7.5, Zn+), and both stresses simultaneously (pH = 7.5, Zn-). Each point represents one gene. LogFC – binary logarithm of expression level fold change; LogFDR – decimal logarithm of the false discovery rate. Genes with expression fold change (increase or decrease) > 2 and FDR < 0.05 are marked with red

Gene expression analysis was performed and genes with expression alterations were identified for each cultivar under the following stresses: 1) Zn deficiency (pH = 5.5, Zn-), 2) unfavorable pH level (pH = 7.5, Zn+), 3) Zn deficiency and unfavorable pH level (pH = 7.5, Zn-). Genes with significant expression alterations were revealed in each analyzed group (Additional files 2, 3, 4, 5, 6, and 7) and visualization of gene expression changes was performed using Volcano plots (Fig. 3) and Venn diagram (Additional file 8). Zn deficiency had fewer effects on expression alterations compared to unfavorable pH and expression changes were relatively similar under unfavorable pH level regardless of Zn content ("pH = 7.5, Zn+" and "pH = 7.5, Zn-" conditions). It should be noted that studied cultivars had diverse expression profiles under the same pH level and Zn content both in control and stress conditions (Fig. 4). This indicates a significant difference between Norlin and Mogilevsky.

### Overrepresented GO terms

For identification of the processes in which up- and down-regulated genes under Zn deficiency, increased pH, or both stresses simultaneously are involved, GO enrichment analysis was performed for individual cultivars.

The following GO terms were overrepresented under Zn deficiency and optimal pH = 5.5: related to oxidoreductase activity – for both cultivars in tops 50/100 upregulated genes; related to photosynthesis – for Norlin in tops 50/100 upregulated genes; related to oxidoreductase activity, photosynthesis, and stress response – for Norlin in tops 300/500 upregulated genes; related to cell wall and antioxidant activity – for Mogilevsky in tops 300/500 upregulated genes; related to cell wall – for Norlin in tops 50/100 and 300/500 downregulated genes; related to oxidoreductase activity – for Mogilevsky in tops 50/100 downregulated genes; related to oxidoreductase activity and photosynthesis – for Mogilevsky in tops of 300/359 downregulated genes (Additional files 9 and 10).

Under control Zn content and pH = 7.5, the following GO terms were overrepresented: related to iron ion homeostasis and vitamin metabolic process – for both cultivars in tops 50/100 upregulated genes; related to heme binding, vitamin metabolic process, iron ion homeostasis, oxidoreductase activity – for both cultivars in tops 300/500 upregulated genes; related to photosynthesis – for Norlin in tops 300/500 upregulated genes; related to extracellular region and heme binding – for Norlin in tops 50/100 and 300/500 downregulated genes; related to oxidoreductase activity and DNA binding – for Mogilevsky in tops 50/100 and 300/500 downregulated genes (Additional files 11 and 12).

Under Zn deficiency and pH = 7.5, the following GO terms were overrepresented: related to zinc ion transmembrane transporter activity – for both cultivars in tops 50/100 upregulated genes (for Norlin FDR was more than 0.05, so overrepresentation was not statistically significant); related to iron ion homeostasis and extracellular region – for Mogilevsky in tops 50/100 upregulated genes; related to response to stress, heme binding, and oxidoreductase activity – for both cultivars in tops 300/500 upregulated genes; related to sulfate transmembrane transporter activity – for Norlin in tops 50/100 downregulated genes; related to cell wall – for both cultivars in tops 300/500 downregulated genes; related to oxidoreductase activity for Mogilevsky in tops 300/500 downregulated genes (Additional files 13 and 14).

Thus, GO terms related to oxidoreductase activity, iron ion homeostasis, cell wall, response to stress, and photosynthesis were the most affected by Zn deficiency and unfavorable pH in flax plants. In tolerant and sensitive cultivars, many processes that were affected by the studied stresses were the same, however, differences in overrepresented categories were also identified. Stimulation of processes involved in photosynthesis was revealed only in tolerant cultivar Norlin in response to Zn deficiency. Possibly, this mechanism provides the tolerance to the stress. Gene expression profiles for GO terms, which were overrepresented under Zn deficiency and pH = 7.5, were visualized by heatmaps and significant differences between studied cultivars were revealed. Heatmap for GO term “response to stress” (ID 0006950) well illustrates diverse gene expression in Norlin and Mogilevsky cultivars both under control and stress conditions and also indicates that unfavorable pH resulted in more significant alterations compared to Zn deficiency (Additional file 15).

## Discussion

Physiological depression of flax plants is associated with non-optimal pH (7.5 or higher) and imbalance of macro- and micronutrients in soil and is often caused by liming [3]. Application of Zn fertilizers is one of the ways to decrease symptoms of physiological depression, however, this measure is not always effective [7]. In the present work, we studied the effects of Zn deficiency, unfavorable pH, and both stresses simultaneously on flax plants with diverse tolerance to physiological depression using transcriptome sequencing.

Under Zn deficiency, inhibition of growth, leaf bronzing, and necrosis were observed for both cultivars - tolerant Norlin and sensitive Mogilevsky. Under pH = 7.5, the same symptoms were much more pronounced, especially in sensitive cultivar Mogilevsky. Effects of both stresses were similar to the effects of pH = 7.5 only. Visualization of expression profiles using MDS plot (Fig. 2), volcano plots (Fig. 3), and heatmaps (Additional file 15) also showed that pH level contribution was more pronounced compared to Zn content. It is known that Zn deficiency can be associated with immobilization of Zn in low available for plant form caused by high soil pH [55–57]. In flax, Zn deficiency had less negative effects on plants, while the increase in pH level results in crucial damages of plants that indicates greater sensitivity of flax to non-optimal soil acidity.

Some similar trends in expression alterations were revealed in examined flax cultivars under the studied stresses. Upregulation of genes encoding metal transporters (ZIP1, ZIP10, NRAMP5, YSL3) was identified under Zn deficiency, pH = 7.5, and both stresses simultaneously in Norlin and Mogilevsky (Additional files 2, 3, 4, 5, 6, and 7). It is known that particular nutrients, including Zn, are less available for plants at neutral and alkaline soils compared to acidic ones [58–61]. Induction of metal transporters, including ZIP, YSL, and NRAMP, under essential metal deficiency was revealed in a number of plant species [22, 62–64]. We observed upregulation of metal transporters in flax under Zn deficiency and unfavorable pH for the first time and it can be assumed that in this way flax plants compensate the effects of low availability of Zn. Overexpression of peroxidase-encoding genes was identified under pH = 7.5 in both cultivars. Peroxidases are antioxidant enzymes, which role in plant stress response is well known [65–67]. Induction of peroxidases in flax plants under unfavorable pH is probably associated with oxidative stress and contributes to detoxification of ROS. Genes with distinct expression changes between examined tolerant and sensitive genotypes were identified. Photosynthesis-related GO terms were overrepresented in top lists of 50/100 upregulated under Zn deficiency genes only in tolerant cultivar Norlin. Zn is an essential element of many proteins, which are involved in numerous biochemical processes [22, 55, 68–70]. It was shown that Zn deficiency results in depression of photosynthesis [71–75]. In flax cultivar Norlin, we revealed the induction of several photosynthesis-related genes under "Zn-" conditions and, possibly in this way, the tolerant genotype overcomes unfavorable effects of Zn deficiency.

It should be noted that tolerant and sensitive cultivars had diverse expression profiles both in control and stress conditions (Fig. 4 and Additional file 15). Therefore, greater tolerance of Norlin cultivar to physiological depression could be associated not only with induction of some genes in response to stress but with constitutive expression of particular genes, which make it more adaptive to the stress.

Thus, expression analysis of genes, which are expressed in flax plants under control conditions and unfavorable pH/Zn deficiency, allowed us to reveal processes that are most affected by the studied stresses. Moreover, identified differences in gene expression between tolerant and sensitive genotypes contribute to the understanding of flax tolerance mechanisms to edaphic stresses.

## Conclusions

We studied the response of flax cultivars with diverse tolerance to unfavorable pH and Zn deficiency using transcriptome sequencing. Under non-optimal pH compared to Zn deficiency, stronger inhibition of plant growth and greater gene expression alterations were revealed for both cultivars. The identified differentially expressed genes are involved in various processes, including ion transport, cell wall biogenesis, oxidoreductase activity, and photosynthesis, and could play an important role in flax response to the studied stresses. Moreover, genes with distinct expression changes between examined tolerant and sensitive genotypes were revealed. These genes could be involved in the mechanisms of flax tolerance to non-optimal soil acidity and/or Zn deficiency.

## Additional files

Additional file 1: Statistics of mapping of Mogilevsky and Norlin reads to Norlin assembly. (XLSX 10 kb)

Additional file 2: Gene expression alterations in flax cultivar Mogilevsky under Zn deficiency. (XLSX 6441 kb)

Additional file 3: Gene expression alterations in flax cultivar Norlin under Zn deficiency. (XLSX 6564 kb)

Additional file 4: Gene expression alterations in flax cultivar Mogilevsky under pH 7.5. (XLSX 6673 kb)

Additional file 5: Gene expression alterations in flax cultivar Norlin under pH 7.5. (XLSX 6700 kb)

Additional file 6: Gene expression alterations in flax cultivar Mogilevsky under Zn deficiency and pH 7.5. (XLSX 6727 kb)

Additional file 7: Gene expression alterations in flax cultivar Norlin under Zn deficiency and pH 7.5. (XLSX 6580 kb)

Additional file 8: Venn diagram illustrating the overlaps of the lists of differentially expressed genes (FDR < 0.05, average CPM > 4) induced by Zn deficiency, unfavorable pH, and both stresses simultaneously in flax cultivars Norlin (tolerant) and Mogilevsky (sensitive). (PNG 1264 kb)

Additional file 9: GO enrichment analysis for tops of up- and down-regulated genes in flax cultivar Mogilevsky under Zn deficiency. (XLSX 10861 kb)

Additional file 10: GO enrichment analysis for tops of up- and down-regulated genes in flax cultivar Norlin under Zn deficiency. (XLSX 12506 kb)

Additional file 11: GO enrichment analysis for tops of up- and down-regulated genes in flax cultivar Mogilevsky under pH 7.5. (XLSX 15825 kb)

Additional file 12: GO enrichment analysis for tops of up- and down-regulated genes in flax cultivar Norlin under pH 7.5. (XLSX 16592 kb)

Additional file 13: GO enrichment analysis for tops of up- and down-regulated genes in flax cultivar Mogilevsky under Zn deficiency and pH 7.5. (XLSX 15771 kb)

Additional file 14: GO enrichment analysis for tops of up- and down-regulated genes in flax cultivar Norlin under Zn deficiency and pH 7.5. (XLSX 12492 kb)

Additional file 15: Patterns of expression of genes participating in response to stress (GO ID 0006950). This heatmap represents Z-scores of normalized read counts per million (CPM) for each gene: from blue (low expression levels) to orange (high expression levels) in flax cultivars Norlin and Mogilevsky. Control conditions are indicated Zn + pH = 5.5; Zn deficiency and optimal pH – Zn- pH = 5.5; optimal Zn content and high pH level – Zn + pH = 7.5; Zn deficiency and high pH level – Zn- pH = 7.5. r1 and r2 – biological replicates. (PNG 864 kb)

## Abbreviations

Blast name: gene name of top BLAST hit(s)
Blast species: organism to which top BLAST hit(s) belongs
Blast symbol: gene symbol of top BLAST hit(s)
FDR: adjusted LRT *p*-value
Gene ID: gene identifier (generated by Trinity)
Gene Ontology: Gene Ontology annotation of the top BLAST hit
KEGG A.Thaliana: KEGG gene/protein identifier (*Arabidopsis thaliana*) of the top BLAST hit
KEGG Orthology: KEGG BRITE gene/protein identifier (pan-organismal) of the top BLAST hit
logCPM: binary logarithm of read counts per million (CPM), average across all the samples
logFC: binary logarithm of gene expression level fold change between control and stress conditions
LR: likelihood ratio from edgeR’s likelihood ratio test (LRT)
Mogilevsky Zn- pH 7.5 r2: CPM per gene in cultivar Mogilevsky under Zn deficiency and unfavorable pH = 7.5 in r2 replicate
Norlin Zn + pH 5.5 r1: CPM per gene in cultivar Norlin under control conditions in r1 replicate
PValue: LRT *p*-value
Transcript lengths: comma-separated lengths of all predicted transcripts
