# Supplementary figures and images for: Flax (Linum usitatissimum L.) response to non-optimal soil acidity and zinc deficiency
Source: BMC Plant Biol. 2019 Feb 15;19(Suppl 1):54. doi: 10.1186/s12870-019-1641-1 (PMC6393972; doi:10.1186/s12870-019-1641-1)

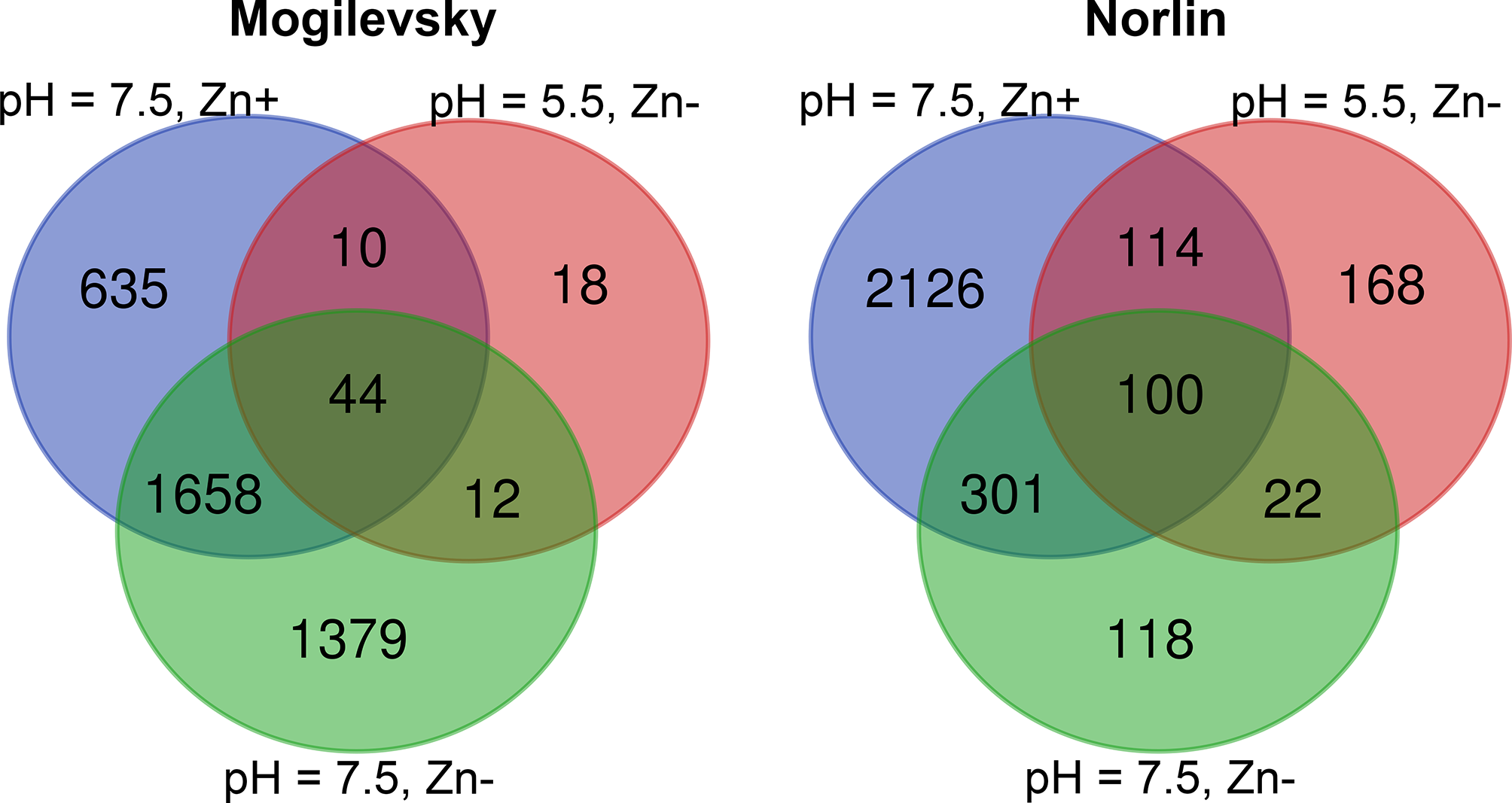

Supplement: Supplementary file 8 — Venn diagram illustrating the overlaps of the lists of differentially expressed genes (FDR < 0.05, average CPM > 4) induced by Zn deficiency, unfavorable pH, and both stresses simultaneously in flax cultivars Norlin (tolerant) and Mogilevsky (sensitive). (PNG 1264 kb) [file 12870_2019_1641_MOESM8_ESM.png]

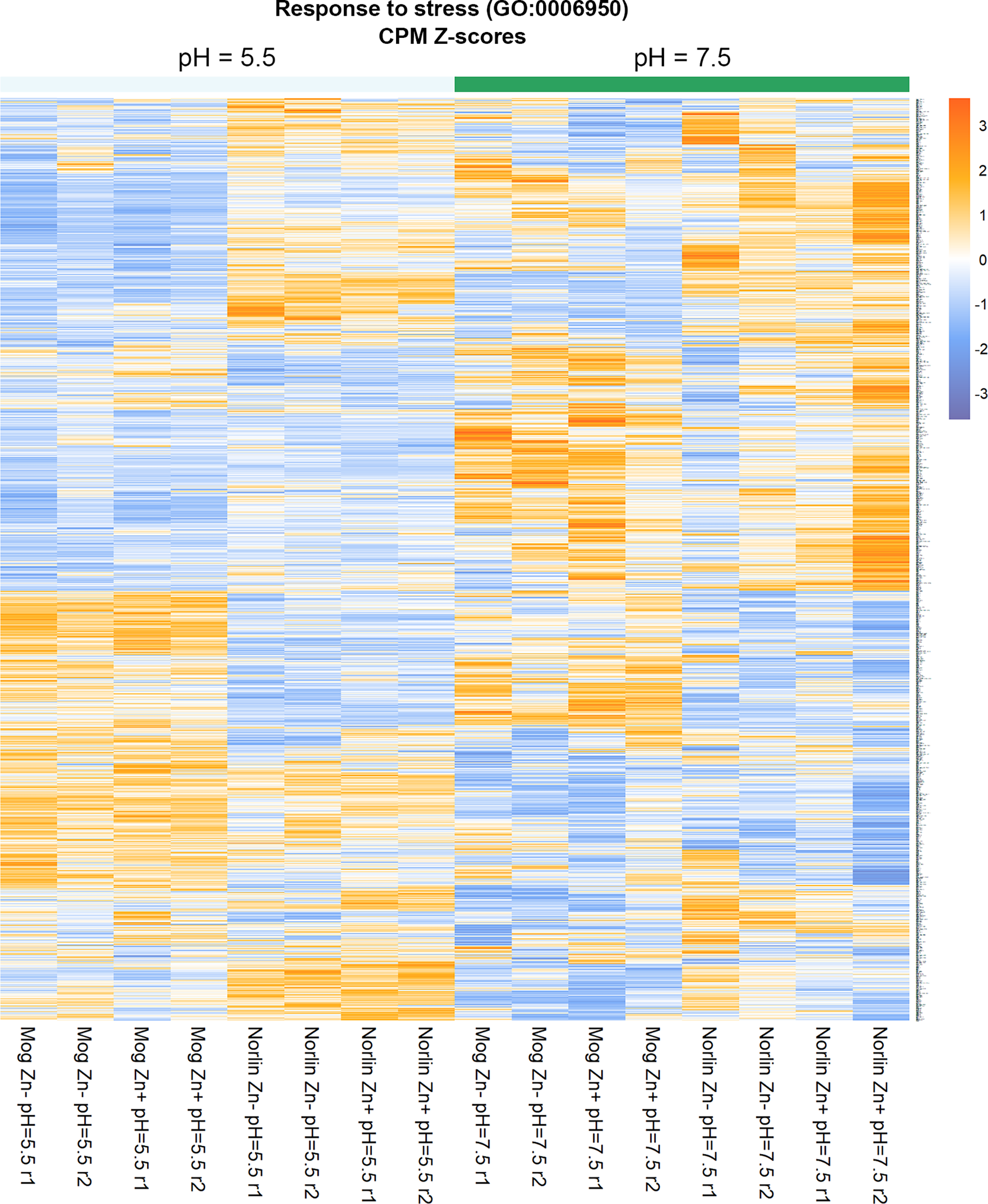

Supplement: Supplementary file 15 — Patterns of expression of genes participating in response to stress (GO ID 0006950). This heatmap represents Z-scores of normalized read counts per million (CPM) for each gene: from blue (low expression levels) to orange (high expression levels) in flax cultivars Norlin and Mogilevsky. Control conditions are indicated Zn + pH = 5.5; Zn deficiency and optimal pH – Zn- pH = 5.5; optimal Zn content and high pH level – Zn + pH = 7.5; Zn deficiency and high pH level – Zn- pH = 7.5. r1 and r2 – biological replicates. (PNG 864 kb) [file 12870_2019_1641_MOESM15_ESM.png]
